# Recipient warm ischemic time negatively influences biliary complications and graft survival – a single center retrospective analysis

**DOI:** 10.3389/fgstr.2025.1601741

**Published:** 2025-08-06

**Authors:** Sophie Reichelt, Alexander Semaan, Philipp Lutz, Jörg C. Kalff, Cornelius J. van Beekum, Steffen Manekeller

**Affiliations:** ^1^ Department of General-, Visceral-, Thoracic- and Vascular Surgery, University Hospital of Bonn, Bonn, Germany; ^2^ Department of Internal Medicine I, University Hospital of Bonn, Bonn, Germany; ^3^ Department of General, Visceral and Transplant Surgery, Hannover Medical School, Hannover, Germany

**Keywords:** liver transplantation, warm ischemia time, biliary complications, biliary stricture, bilirubin, organ donation, graft function

## Abstract

Recipient warm ischemia time (rWIT) in liver transplantation (LT) – which is defined as the time from removal of the graft from cold storage until reperfusion with portal and/or arterial blood flow – has been linked to negative outcomes. Biliary complications, particularly biliary strictures, are a major cause of morbidity after LT. However, the relationship between rWIT in donation after brain death (DBD) LT and biliary strictures has not been well explored. This single-center study retrospectively analyzed data from 162 DBD-LT recipients (2013-2022). Patients were divided into two groups: rWIT ≤30 minutes (n=33) and rWIT >30 minutes (n=129). Livers did not undergo any *in situ* or *ex situ* machine perfusion techniques. Biliary complications occurred at similar rates in both groups (p=0.5). Biliary strictures tended to be more common in the rWIT >30 minutes group, although without statistical significance (40% vs. 24%; p=0.1). The median serum bilirubin levels on day 5 were significantly higher in the rWIT >30-minute group (5.2 (IQR 2.6, 8.9) mg/dl vs. 3.7 (IQR 1.9, 5.9) mg/dl; p=0.013). Patients with rWIT >30 minutes required significantly more blood transfusions intraoperatively (p=0.021). There was a high tendency for higher severe complication rates in the rWIT >30-minute group, which was not significant (58% vs. 39%; p=0.054). Prolonged rWIT in LT was associated with a trend toward a higher incidence of bile duct strictures and elevated liver enzymes. However, due to the retrospective design and risk of selection bias, rWIT should be interpreted as one of several contributing factors. Our findings suggest that minimizing rWIT may support better outcomes, but causality cannot be definitively established.

## Introduction

Liver transplantation (LT) is frequently the only curative option for patients with severe liver conditions such as alcohol-related liver disease, primary sclerosing cholangitis, certain liver tumors, and acute liver failure. One of the key factors affecting the success of a liver transplant is ischemia time, which consists of two main components: cold ischemia time (CIT) and recipient warm ischemia time (rWIT).

CIT denotes the interval between the initiation of cold preservation, marked by perfusion of the donor liver with a preservation solution and the removal of the graft from cold storage immediately prior to implantation. rWIT, on the other hand, occurs after the liver is removed from cold storage and before blood flow is restored through portal or arterial reperfusion once the liver is transplanted into the recipient. This time period is especially critical for livers from donors after brain death.

Prolonged ischemia times, both CIT and WIT, are strongly associated with several adverse post-transplant outcomes, including early allograft dysfunction, graft loss, ischemic cholangiopathy (a form of bile duct injury), and prolonged hospital stays (1–8). Reducing ischemia time is therefore an important strategy in optimizing LT outcomes.

It has been suggested that a rWIT of ≤30 minutes has a positive prognostic impact. However, studies focusing on rWIT in donation after brain death (DBD) are less common compared to those examining donor WIT in donation after circulatory death (DCD) (5, 6, 9).

Biliary complications are among the most common and significant post-transplant complications in LT. These complications occur in 10 to 40% of transplant recipients (10, 11) and can significantly impact outcomes, contributing to graft loss, increased mortality, and the need for re-transplantation (12–14). Previous studies have shown a lower rate of biliary complications in DBD organs compared to DCD organs (15, 16).

Biliary strictures—narrowing of the bile ducts—are a major type of biliary complication and are classified into two main types: anastomotic (occurring at the site of the bile duct anastomosis) and non-anastomotic (occurring in other parts of the bile duct) (13, 17). Both types can occur simultaneously. Anastomotic strictures are more common and are typically due to technical issues during surgery, while non-anastomotic strictures may be related to ischemia or other factors related to the liver transplant process.

Treatment options for biliary strictures include:

Endoscopic retrograde cholangiopancreatography (ERCP), which allows for direct visualization of the bile ducts and can be used to perform therapeutic interventions such as balloon dilation or stent insertion to open up narrowed areas (12, 18).

Percutaneous transhepatic cholangiography is a technique that involves inserting a catheter through the skin into the liver to drain bile from blocked ducts (12, 18). Percutaneous transhepatic cholangial drainage (PTCD) is often used when endoscopic treatments are not feasible or effective.

Despite these treatment options, biliary complications remain a significant challenge in LT, as they can lead to worsened graft function and the need for further interventions (10, 13, 14, 19). Early diagnosis and appropriate management are critical in improving transplant outcomes.

Very few studies focus on the relationship between rWIT and biliary complications. The association of prolonged rWIT with post-transplant biliary strictures in living donor LT is described by Sakamoto et al. (20). Welling et al. proclaim that rWIT influences bile leakage, which in turn promotes bile duct strictures (11). Nonanastomotic biliary strictures during the first year after LT correlate with a prolonged rWIT, as Buis et al. demonstrate (14).

The current body of research highlights the significant impact of prolonged rWIT on biliary complications in LT, but much of the focus has historically been on donor WIT in DCD. In contrast, studies specifically addressing rWIT in DBD LT are still relatively limited. Furthermore, there is a scarcity of research that specifically targets the development of biliary strictures in the context of prolonged rWIT.

Our retrospective study aims to fill the gap in the literature by examining the link between prolonged rWIT and biliary complications, especially bile duct strictures, in DBD-LT. Biliary strictures are a common complication after LT and can significantly affect both graft function and patient survival. By studying this relationship, our research may help determine whether longer rWIT is an independent risk factor for developing strictures, which could guide clinical practices in managing donors and recipients in LT.

## Materials and methods

### Patient selection and data acquisition

This retrospective, single-center study included 162 patients who underwent LT between 2013 and 2022 in the Department of General-, Visceral-, Thoracic- and Vascular Surgery at the University Hospital of Bonn. All organs were obtained from DBD. Patients under the age of 18, those who had undergone a transplant with the use of a machine perfusion system and retransplantations were excluded from the study. All transplants were performed by highly experienced transplant and organ recovery surgeons. The outcomes were followed up over a period up to 11 years. All patients fulfilled the Eurotransplant eligibility criteria. The electronic patient database was employed for the acquisition of data. The study was conducted in accordance with the tenets of the Declaration of Helsinki.

### Definitions

In our study, recipient Warm Ischemia Time (rWIT) is defined as the interval between removing the graft from cold storage and achieving reperfusion via portal and/or hepatic arterial anastomosis. This period is critical because prolonged rWIT can contribute to liver injury and complications, such as biliary strictures.

Cold Ischemia Time (CIT), on the other hand, refers to the duration between the initiation of the cold perfusion in the donor and the removal of the graft from the cold preservation solution before implantation into the recipient. While both CIT and rWIT are important factors influencing transplant outcomes, rWIT is particularly relevant to the recipient’s experience and graft viability.

Arterial anastomosis time describes the period from portal reperfusion to arterial reperfusion.

### Surgical technique

All liver transplantations were performed using a standardized surgical approach. The piggyback technique was used in all cases. Vascular reconstruction followed a uniform sequence, starting with portal vein anastomosis, followed by hepatic artery anastomosis. Biliary reconstruction was performed using an end-to-end duct-to-duct anastomosis in all patients, without the use of T-tubes or external biliary drains.

#### Surgical complications and classification

During the hospital stay, surgical complications were evaluated using the Clavien-Dindo classification, which categorizes complications based on the severity of treatment required (21):

Grade I involves complications that do not require any invasive treatment.
Grade II involves complications that require medication.
Grade III involves complications requiring surgical, endoscopic, or radiological interventions, with regional/local anesthesia (Grade IIIa) or with general anesthesia (Grade IIIb).
Grade IV involves life-threatening complications requiring intensive care.
Grade V refers to death.

#### Arterial and vascular complications

An arterial complication refers to hemorrhage or occlusion of an arterial liver vessel. The term vascular occlusion is used to describe the obstruction of any arterial, venous, or portal venous liver vessel, which can lead to poor graft function or failure if not promptly managed.

#### Primary graft dysfunction and primary graft non-function

Primary graft dysfunction was defined according to the criteria proposed by Olthoff et al. which include any of the following within the first 7 days post-transplant (32):

- Bilirubin ≥10 mg/dL on postoperative day 7,
- International normalized ratio (INR) ≥1.6 on postoperative day 7, or
- Peak alanine aminotransferase (ALT) or aspartate aminotransferase (AST) >2,000 IU/L.

When the graft dysfunction progresses into the need for retransplantation or progression to death due to graft nonfunction are defined as primary graft non-function (22, 32).

### Statistical analysis

All tables, statistical calculations and figures were created with R version 4.2.1 and R Studio version 2022.07.2 for Windows (R Foundation for Statistical Computing, Vienna, Austria) using the packages tidyverse, gtsummary, survminer and survival. A p-value of < 0.05 was defined statistically significant. The data were presented as median and interquartile range. To compare and illustrate differences in graft survival, univariate analysis and Kaplan-Meier survival analysis with the log-rank test were performed.

## Results

### Recipient, donor and transplant characteristics

The study included 162 liver transplant recipients. Of these, 67 (41%) were female and 95 (59%) were male. The median age was 56 (IQR 48, 63) years. The most common primary liver diseases were alcohol-related liver disease in 39 (24%), primary sclerosing cholangitis in 21 (13%), Hepatitis C in 19 (12%) and cryptogenic liver cirrhosis in 19 (12%) transplant recipients. Hepatocellular carcinoma occurred in 42 (26%) patients. The median laboratory Model of End Stage Liver Disease (MELD) score at transplant was 24 (IQR 10, 34) and 21 (13%) recipients had high-urgency status. The median operation time was 312 (IQR 266, 375) min, the median CIT was 544 (IQR 468, 615) min, and the median rWIT was 38 (IQR 32, 46) min. A median of 6 (IQR 2, 12) units of RBCs, 8 (IQR 4, 16) units of FFPs and 2 (IQR 0, 4) units of platelets were transfused intraoperatively. Transplant patients were divided into two groups, one group with a rWIT of 30 min or less and another group with a rWIT of more than 30 min. There were 33 patients in the rWIT ≤30 min group and 129 patients in the rWIT >30 min group. Differences in recipients or donor characteristics were seen in preoperative inserted transjugular intrahepatic portosytemic shunt (9% in the rWIT ≤30 min group versus 33% in the rWIT >30 min group, p-value 0.042) as well as in the donor liver weight (1452 (IQR 1200, 1558) g versus 1680 (IQR 1370, 1910) g, p value 0.001). In the rWIT >30 min group, significantly more blood products were transfused intraoperatively (for RBC, FFP and platelet transfusions p-values 0.021, 0.006 and 0.045). **Table 1** contains comprehensive information on recipients, donors and transplantation data.

**Table 1:** Recipient, donor and transplant characteristics.

Characteristic	Overall N = 162*^1^*	WIT ≤ 30min N = 33*^1^*	WIT > 30min N = 129*^1^*	p-value*^2^*
Recipient characteristic				
Recipient sex ratio (F:M)	67 (41%): 95 (59%)	17 (52%): 16 (48%)	50 (39%): 79 (61%)	0.2
Recipient age (y)	56 (48, 63)	53 (47, 61)	56 (49, 63)	0.5
Recipient BMI (kg/m²)	25.5 (22.9, 28.1)	25.6 (22.0, 28.0)	25.4 (23.0, 28.1)	>0.9
Primary liver disease				
Alcoholic liver disease	39 (24%)	8 (24%)	31 (24%)	
PSC	21 (13%)	11 (33%)	10 (7.8%)	
Hepatitis C	19 (12%)	1 (3.0%)	18 (14%)	
Cryptogenic liver cirrhosis	19 (12%)	4 (12%)	15 (12%)	
Toxic hepatitis	11 (6.8%)	1 (3.0%)	10 (7.8%)	
Hepatitis B	11 (6.8%)	2 (6.1%)	9 (7.0%)	
NASH	7 (4.3%)	1 (3.0%)	6 (4.7%)	
Primary biliary cirrhosis	4 (2.5%)	0 (0%)	4 (3.1%)	
Autoimmune hepatitis	3 (1.9%)	0 (0%)	3 (2.3%)	
Others	28 (17%)	5 (15%)	23 (18%)	
HCC	42 (26%)	9 (27%)	33 (26%)	0.8
Laboratory MELD score at transplant	24 (10, 34)	17 (8, 32)	28 (12, 34)	0.067
High-urgency status	21 (13%)	4 (12%)	17 (13%)	>0.9
Child Pugh Score				0.9
A	43 (27%)	10 (30%)	33 (26%)	
B	51 (31%)	10 (30%)	41 (32%)	
C	68 (42%)	13 (39%)	55 (43%)	
Comorbidities				
Renal	74 (46%)	12 (36%)	62 (48%)	0.2
Kardial	30 (19%)	4 (12%)	26 (20%)	0.3
Diabetes mellitus	38 (23%)	7 (21%)	31 (24%)	0.7
PAH	25 (15%)	2 (6.1%)	23 (18%)	0.095
TIPS	36 (22%)	3 (9.1%)	33 (26%)	0.042*
Previous abdominal surgery	67 (41%)	17 (52%)	50 (39%)	0.2
Donor characteristics				
Donor age (y)	52 (40, 67)	50 (40, 66)	53 (41, 67)	0.6
Donor liver weight (g)	1600 (1340, 1890)	1452 (1200, 1558)	1680 (1370, 1910)	0.001*
Transplantation characteristics				
Operation time (min)	312 (266, 375)	276 (237, 317)	322 (275, 385)	0.001*
Cold ischemia time (min)	544 (468, 615)	574 (470, 653)	540 (468, 602)	0.2
Arterial anastomosis time (min)	34 (29, 42)	25 (23, 27)	37 (32, 46)	<0.001*
RBC transfusions (units)	6 (2, 12)	4 (0, 10)	7 (3, 13)	0.021*
FFP transfusions (units)	8 (4, 16)	6 (2, 10)	10 (6, 17)	0.006*
Platelet transfusions (units)	2 (0, 4)	0 (0, 2)	2 (0, 4)	0.045*

*^1^* n (%); Median (IQR).

*^2^* Pearson's Chi-squared test; Wilcoxon rank sum test; Fisher's exact test.

*p-Value < 0.05 represents significant results.

BMI, body mass index; FFP, fresh frozen plasma; min, minutes; HCC, hepatocellular carcinoma; MELD, model of end stage liver disease; NASH, non-alcoholic steatohepatitis; PAH, Pulmonary arterial hypertension; PSC, primary sclerosing cholangitis; RBC, red blood cell; TIPS, transjugular intrahepatic portosystemic shunt; y, years.

### Perioperative outcome depending on rWIT with a cut-off of 30 minutes

The perioperative outcome was considered on the basis of the two groups, rWIT ≤30 min and rWIT >30 min. There was no difference between the groups regarding biliary complications (p-value 0.5), ERCP or PTCD insertion (p-value 0.6), whereas bile duct strictures were more frequent in rWIT >30 min group (40%) than in rWIT ≤30 min group (24%) (p-value 0.1). Bile leakage was less often detected in rWIT >30 min group (7% versus 21%, p-value 0.023). The median serum bilirubin at day 5 was significantly higher in rWIT >30 min group with a value of 5.2 (IQR 2.6, 8.9) mg/dl compared to 3.7 (IQR 1.9, 5.9) mg/dl in the other group with a p-value of 0.013. There was a trend towards more major complications in the rWIT >30 min group compared to the rWIT ≤30 min group (p-value 0.054). Primary nonfunction occurred significantly more often in rWIT >30 min group with 17 cases (13%), whereas none occurred in rWIT ≤30 min group (p-value 0.025). The outcome data stratified by rWIT are comprised in **Table 2**.

**Table 2:** Outcome data depending on WIT with a cut-off of 30 minutes.

Outcome	Overall N = 162*^1^*	WIT ≤ 30min N = 33*^1^*	WIT > 30min N = 129*^1^*	p-value*^2^*
Clavien Dindo ≥ IIIb	88 (54%)	13 (39%)	75 (58%)	0.054
Relaparotomy	50 (31%)	8 (24%)	42 (33%)	0.4
Postoperative bleeding	33 (20%)	5 (15%)	28 (22%)	0.4
Primary nonfunction	17 (10%)	0 (0%)	17 (13%)	0.025*
Rejection	17 (10%)	4 (12%)	13 (10%)	0.8
Necessity of retransplantation	16 (9.9%)	2 (6.1%)	14 (11%)	0.5
Biliary complication	68 (42%)	12 (36%)	56 (43%)	0.5
Bile leakage	16 (9.9%)	7 (21%)	9 (7.0%)	0.023*
Bile duct stricture	59 (36%)	8 (24%)	51 (40%)	0.10
Biliary anastomotic stricture	50 (83%)	6 (75%)	44 (85%)	
ERCP or PTCD	70 (43%)	13 (39%)	57 (44%)	0.6
Bilirubin day 5 (mg/dl)	4.7 (2.5, 8.3)	3.7 (1.9, 5.9)	5.2 (2.6, 8.9)	0.013*
Bilirubin peak within 30 days (mg/dl)	10 (6, 17)	9 (5, 14)	10 (6, 18)	0.4
Arterial complication	9 (5.6%)	2 (6.1%)	7 (5.4%)	>0.9
Vascular occlusion	13 (8.0%)	2 (6.1%)	11 (8.5%)	>0.9
Postoperative wound infection	18 (11%)	2 (6.1%)	16 (12%)	0.5
Pneumonia	38 (23%)	5 (15%)	33 (26%)	0.2
ICU stay (d)	7 (3, 22)	6 (3, 18)	7 (3, 22)	0.8
Hospital stay (d)	26 (17, 52)	29 (18, 50)	25 (17, 52)	>0.9
1-year graft loss	44 (27%)	7 (21%)	37 (29%)	0.4

*^1^* n (%); Median (IQR).

*^2^* Pearson's Chi-squared test; Wilcoxon rank sum test; Fisher's exact test.

*p-Value < 0.05 represents significant results.

d, days; ERCP, endoscopic retrograde cholangiopancreatography; ICU, intensive care unit; PTCD, percutaneous transhepatic cholangio drain.

**Figure 1:**
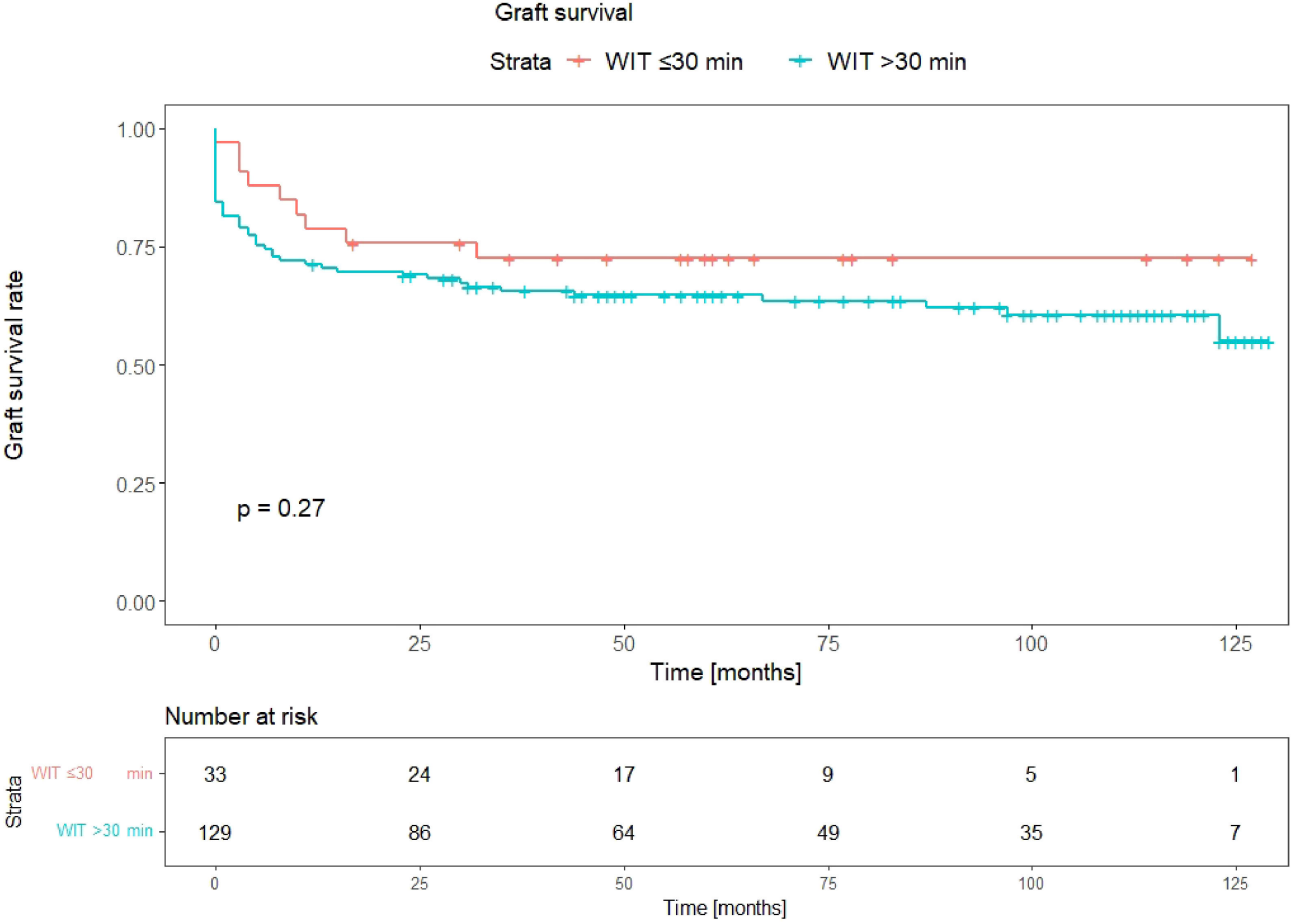
Kaplan-Meier curves depicting graft survival for WIT ≤30min compared to WIT >30min. p-value: log-rank comparison of survival curves.

The graft survival rate was lower for transplants with a rWIT >30 min than for transplants with rWIT ≤30 min, albeit without significance (p-value 0.27). This is illustrated as a Kaplan-Meier curve in **Figure 1**.

### Perioperative outcome depending on four rWIT groups: ≤30 min, 31–40 min, 41–50 min, >50 min

In order to investigate whether biliary complications have a different frequency depending on the rWIT time, the patients were divided into four groups: rWIT ≤30 min (n=33), rWIT 31–40 min (n=65), rWIT 41–50 min (n=35) and rWIT >50 min (n=29). As before, the groups showed no significant difference in terms of biliary complications (p-value >0.9) and ERCP or PTCD insertion (p-value 0.9). In the groups with increasing rWIT, bile duct strictures had an upward trend (24%, 38%, 40%, 41%, p-value 0.4). The median bilirubin value on day 5 also showed an upward trend in the four groups (3.7 (IQR 1.9, 5.9), 4.9 (IQR 2.8, 9.4), 5.2 (IQR 2.6, 8.7), 5.5 (IQR 3.1, 7.6) mg/dl, p-value 0.1). The detailed data are listed in **Table 3**.

**Table 3:** Outcome data of the respective WIT groups.

Characteristic	≤30 min N = 33*^1^*	31–40 min N = 65*^1^*	41–50 min N = 35*^1^*	>50 min N = 29*^1^*	*p*-value*^2^*
Intraoperative RBC transfusions (units)	4 (0, 10)	7 (3, 13)	6 (2, 11)	9 (5, 20)	0.019*
Intraoperative FFP transfusions (units)	6 (2, 10)	9 (6, 16)	8 (4, 14)	18 (8, 27)	0.003*
Intraoperative platelet transfusions (units)	0 (0, 2)	2 (0, 4)	2 (1, 4)	4 (2, 6)	<0.001*
Operation time (min)	276 (237, 317)	285 (250, 324)	358 (300, 396)	411 (353, 442)	<0.001*
Cold ischemia time (min)	574 (470, 653)	534 (468, 592)	538 (450, 620)	565 (487, 630)	0.4
Arterial nastomosis time (min)	25 (23, 27)	32 (30, 34)	40 (39, 43)	53 (50, 58)	<0.001*
Clavien Dindo ≥ IIIb	13 (39%)	39 (60%)	19 (54%)	17 (59%)	0.3
Relaparotomy	8 (24%)	21 (32%)	11 (31%)	10 (34%)	0.8
Postoperative bleeding	5 (15%)	18 (28%)	5 (14%)	5 (17%)	0.3
Primary nonfunction	0 (0%)	9 (14%)	3 (8.6%)	5 (17%)	0.062
Rejection	4 (12%)	6 (9.2%)	2 (5.7%)	5 (17%)	0.5
Necessity of retransplantation	2 (6.1%)	6 (9.2%)	4 (11%)	4 (14%)	0.8
Biliary complication	12 (36%)	28 (43%)	15 (43%)	13 (45%)	>0.9
Bile leakage	7 (21%)	6 (9.2%)	3 (8.6%)	0 (0%)	0.044*
Bile duct stricture	8 (24%)	25 (38%)	14 (40%)	12 (41%)	0.4
Biliary anastomotic stricture	6 (75%)	25 (96%)	11 (79%)	8 (67%)	0.049*
ERCP or PTCD	13 (39%)	28 (43%)	17 (49%)	12 (41%)	0.9
Bilirubin day 5 (mg/dl)	3.7 (1.9, 5.9)	4.9 (2.8, 9.4)	5.2 (2.6, 8.7)	5.5 (3.1, 7.6)	0.10
Bilirubin peak within 30 days (mg/dl)	9 (5, 14)	9 (5, 15)	11 (7, 22)	11 (8, 15)	0.4
Arterial complication	2 (6.1%)	4 (6.2%)	2 (5.7%)	1 (3.4%)	>0.9
Vascular occlusion	2 (6.1%)	6 (9.2%)	2 (5.7%)	3 (10%)	0.9
Postoperative wound infection	2 (6.1%)	3 (4.6%)	6 (17%)	7 (24%)	0.019*
Pneumonia	5 (15%)	12 (18%)	9 (26%)	12 (41%)	0.059
ICU stay (d)	6 (3, 18)	6 (3, 16)	7 (3, 28)	7 (4, 25)	0.7
Hospital stay (d)	29 (18, 50)	24 (16, 49)	26 (19, 54)	28 (20, 50)	0.8
1-year graft loss	7 (21%)	26 (40%)	4 (11%)	7 (24%)	0.015*

*^1^* Median (IQR); n (%).

*^2^* Kruskal-Wallis rank sum test; Pearson's Chi-squared test; Fisher's exact test.

* *p*-Value < 0.05 represents significant results.

d, days; ERCP, endoscopic retrograde cholangiopancreatography; ICU, intensive care unit; PTCD, percutaneous transhepatic cholangio drain.

### Outcome data in the context of influencing factors – univariate and multivariate analysis

**Table 2** presents an overview of postoperative outcomes. Biliary complications were observed in 68 patients (42%), including bile leakage in 16 cases (10%) and biliary strictures in 59 cases (36%).

**Table 4** provides a detailed analysis of postoperative complications in relation to potential perioperative risk factors. A serum bilirubin level greater than 4.7 mg/dl on postoperative day 5—corresponding to the cohort median—was significantly associated with higher pretransplant MELD scores and increased intraoperative RBC transfusion requirements. One-year graft loss also showed a significant correlation with the volume of intraoperative transfusions. Major complications (Clavien-Dindo grade IIIb or higher) were associated with higher MELD scores, renal and cardiac comorbidities, presence of a TIPS, and transfusion burden. Bile leakage was significantly associated with rWIT less than 30 minutes and showed trends toward lower BMI and prior abdominal surgery. Intraoperative RBC transfusion emerged as a consistent predictor of multiple adverse outcomes, including elevated bilirubin, graft loss, major complications, primary nonfunction, and rejection. Primary nonfunction was further associated with prolonged operative time and rWIT greater than 30 minutes. Graft rejection correlated with longer cold ischemia time and transfusion requirement.

**Table 4:** Occurrence of various risk factors for certain outcomes.

Characteristic	Biliary complication N=68; *p*-value	Bile duct stricture N=59; *p*-value	Bilirubin day 5 >4.7 mg/dl N=80; *p*-value	Bile leakage N=16; *p*-value	1-year graft loss N=44; *p*-value	Clavien Dindo ≥ IIIb N=88; *p*-value	Primary nonfunction N=17; *p*-value	Rejection N=17; *p*-value
Age	56 (50,63); 0.4	57 (51, 63); 0.3	55 (50, 60); 0.5	51 (44, 61); 0.4	55 (49, 61); 0.7	56 (49, 62); 0.7	52 (40, 60); 0.2	54 (49, 57); 0.2
BMI	25.4 (23.0, 28.0); 0.7	25.1 (22.7, 27.5); 0.3	24.8 (22.6, 28.1); 0.6	23.5 (19.7, 26.3); 0.065	25.7 (23.8, 28.0); 0.6	25.1 (22.8, 28.1); 0.6	24.8 (23.7, 28.1); 0.7	25.6 (23.0, 28.7); 0.8
MELD	28 (12, 36); 0.2	30 (12, 36); 0.087	30 (13, 37); 0.002*	17 (10, 32); 0.3	32 (12, 36); 0.095	32 (16, 36); <0.001*	17 (8, 38); 0.8	19 (12, 30); 0.4
Renal	35 (51%); 0,2	31 (53%); 0.2	36 (45%); 0.9	9 (56%); 0.4	23 (52%); 0.3	49 (56%); 0.005*	6 (35%); 0.4	6 (35%); 0.4
Diabetes mellitus	19 (28%); 0.3	16 (27%); 0.4	16 (20%); 0.3	2 (12%); 0.4	8 (18%); 0.3	18 (20%); 0.3	5 (29%); 0.6	2 (12%); 0.4
Cardiac	13 (19%); 0.9	12 (20%); 0.7	13 (16%); 0.4	3 (19%); 0.9	10 (23%); 0.4	22 (25%); 0.021*	2 (12%); 0.7	4 (24%); 0.5
PAH	13 (19%); 0.3	13 (22%); 0.078	11 (14%); 0.5	3 (19%); 0.7	5 (11%); 0.4	17 (19%); 0.14	1 (5.9%); 0.5	3 (18%); 0.7
TIPS	19 (28%); 0.1	17 (29%); 0.1	22 (28%); 0.1	4 (25%); 0.8	12 (27%); 0.3	25 (28%); 0.039*	4 (24%); 0.9	1 (5.9%); 0.12
Abdominal surgery	32 (47%); 0.2	27 (46%); 0.4	30 (38%); 0.3	10 (62%); 0.070	20 (45%); 0.5	34 (39%) 0.4	8 (47%); 0.6	5 (29%); 0.3
Operation time (min)	308 (274, 377); 0.9	309 (276, 394); 0.6	316 (274, 392); 0.2	295 (258, 333); 0.2	314 (281, 361); 0.6	320 (276, 392); 0.055	347 (312, 415); 0.029*	343 (295, 374); 0.5
CIT (min)	544 (470, 624); 0.5	537 (466, 600); 0.9	554 (493, 620); 0.2	577 (498, 689); 0.3	548 (504, 625); 0.5	536 (480, 622); 0.8	559 (517, 641); 0.2	598 (544, 640); 0.037*
WIT>30min	56 (82%); 0.6	51 (86%); 0.1	68 (85%); 0.079	9 (56%); 0.023*	37 (84%); 0.4	75 (85%); 0.054	17 (100%); 0.025*	13 (76%); 0.8
Arterial anastomosis time	34 (29,44); 0.6	35 (30, 46); 0.12	34 (30, 44); 0.4	29 (25, 35); 0.006*	33 (30, 37); 0.6	35 (31, 42); 0.1	36 (33, 50); 0.093	32 (30, 42); 0.7
RBC transfusion (units)	6 (3, 12); 0.8	6 (4, 12); 0.4	8 (4, 16); 0.001*	8 (4, 13); 0.6	11 (6, 16); 0.001*	10 (6, 16); <0.001*	12 (8, 28); 0.012*	4 (2, 5); 0.012*

*^1^* Median (IQR); n (%).

*^2^* Wilcoxon rank sum test; Pearson's Chi-squared test; Fisher's exact test.

* *p*-Value < 0.05 represents significant results.

BMI, body mass index; CIT, cold ischemia time; MELD, model for end-stage liver disease; PAH, pulmonary arterial hypertension; RBC, red blood cells; TIPS, transjugular intrahepatic portosystemic shunt; WIT, warm ischemia time; RBC, red blood cells.

**Table 5** presents multivariate linear regression models for postoperative bilirubin, bile duct stricture, and primary nonfunction. Elevated bilirubin remained independently associated with rWIT greater than 30 minutes (p = 0.0397) and intraoperative transfusions (p = 0.002).

**Table 5:** Potential risk factors for elevated bilirubin at day 5, bile duct stricture and primary nonfunction.

Risk factor	Coefficient (β)	Standard Error	t-value	*p*-value
Bilirubin day 5 (Multiple R-squared: 0.1597, Adjusted R-squared: 0.1259)				
Intercept	3.629	2.273	1.597	0.112
WIT > 30min	2.230	1.0565	2.112	0.036*
CIT	0.002	0.003	0.639	0.524
Operation time	-0.006	0.006	-0.973	0.332
Arterial anastomosis time	-0.030	0.045	-0.673	0.502
Intraoperative RBC transfusion	0.157	0.052	3.033	0.003*
MELD score	0.052	0.036	1.470	0.143
Bile duct stricture (Multiple R-squared: 0.0409, Adjusted R-squared: 0.00279)				
Intercept	0.053	0.244	0.218	0.827
WIT > 30min	0.064	0.114	0.557	0.578
CIT	0.0002	0.0003	0.629	0.530
Operation time	-0.0006	0.0007	-0.916	0.361
Arterial anastomosis time	0.008	0.005	1.387	0.167
Intraoperative RBC transfusion	0.002	0.005	0.367	0.714
MELD score	0.004	0.004	1.014	0.312
Primary nonfunction (Multiple R-squared: 0.1265, Adjusted R-squared: 0.09182)				
Intercept	-0.100	0.145	-0.690	0.491
WIT > 30min	0.110	0.068	1.608	0.110
CIT	0.0003	0.0002	1.680	0.095
Operation time	-0.0004	0.0004	-0.928	0.355
Arterial anastomosis time	0.002	0.003	0.521	0.603
Intraoperative RBC transfusion	0.011	0.003	3.459	0.0007*
MELD score	-0.005	0.002	-2.132	0.035*

Multiple linear regression analysis.

*p-Value < 0.05 represents significant results.

CIT, cold ischemia time; MELD, model for end-stage liver disease; RBC, red blood cells; WIT, warm ischemia time.

**Table 6** summarizes the multivariate analysis of graft survival including rWIT, CIT, operative and arterial anastomosis time, MELD score, and comorbidities. rWIT greater than 30 minutes was associated with increased risk of graft loss (hazard ratio 1.820).

**Table 6:** Multivariate analysis of potential risk factors for graft survival.

Risk factor	HR	95% CI of HR	*p*-value
WIT > 30 min	1.820	0.742- 4.463	0.191
CIT	1.002	0.999 -1.004	0.198
Operation time	0.999	0.993-1.002	0.562
Arterial anastomosis time	0.988	0.948- 1.029	0.556
Intraoperative RBC transfusion	1.013	0.980-1.047	0.447
MELD score	1.015	0.980-1.045	0.304
Renal comorbidity	0.962	0.534 - 1.733	0.896
Diabetes mellitus	0.771	0.383 - 1.551	0.466
cardiac comorbidity	1.542	0.809 - 2.939	0.188
Pulmonary arterial hypertension	0.881	0.411 - 1.890	0.746
TIPS	1.305	0.687 -2.490	2.478
Previous abdominal surgery	1.222	0.692 - 2.157	0.488
Bilirubin day 5 >4.7 mg/dl	1.154	0.635 – 2.099	0.638

Cox proportional hazard regression model.

CIT, cold ischemia time; MELD, model for end-stage liver disease; RBC, red blood cells; TIPS, transjugular intrahepatic portosystemic shunt; WIT, warm ischemia time.

**Table 7** demonstrates the correlation of serum bilirubin > the median 4.7 mg/dl with several complications, including severe postoperative complications (≥ Clavien-Dindo IIIb) (p value 0.011), primary nonfunction (p value 0.015), biliary complications (p value 0.011), bile duct strictures (p value 0.002), the need for ERCP or PTCD (p value 0.0004), as well as prolonged ICU (p value 0.005) and hospital stays (p value 0.008).

**Table 7:** Outcomes depending on Bilirubin on the 5th postoperative day > 4.7 mg/dl.

Characteristic	Bilirubin day 5 ≤ 4.7 mg/dl N = 80*^1^*	Bilirubin day 5 > 4.7 mg/dl N = 80*^1^*	p-value*^2^*
Clavien Dindo ≥ IIIb	35 (44%)	51 (64%)	0.011*
Primary nonfunction	3 (3.8%)	12 (15%)	0.015*
Rejection	11 (14%)	6 (7.5%)	0.2
Re-LTX	5 (6.2%)	11 (14%)	0.11
Biliary complication	26 (32%)	42 (52%)	0.011*
Bile leakage	8 (10%)	8 (10%)	>0.9
Bile duct stricture	20 (25%)	39 (49%)	0.002*
ERCP or PTCD	26 (32%)	44 (55%)	0.004*
Arterial complication	3 (3.8%)	6 (7.5%)	0.5
Vascular occlusion	4 (5.0%)	9 (11%)	0.15
Pneumonia	13 (16%)	25 (31%)	0.026*
ICU stay (d)	5 (3, 12)	9 (4, 34)	0.005*
Hospital stay (d)	22 (16, 42)	34 (21, 67)	0.008*
1-year graft loss	17 (21%)	25 (31%)	0.2

*^1^* Median (IQR); n (%).

*^2^* Wilcoxon rank sum test; Pearson's Chi-squared test; Fisher's exact test.

*p-Value < 0.05 represents significant results.

d, days; ERCP, endoscopic retrograde cholangiopancreatography; ICU, intensive care unit; PTCD, percutaneous transhepatic cholangio drain.

## Discussion

Despite improved overall liver transplant outcomes, post-transplant biliary complications remain a significant cause of morbidity. Biliary complications comprise non-anastomotic biliary strictures, anastomotic leakage/stenosis and ampullary dysfunction. All remain a significant challenge in LT with limited treatment options and a frequent necessity for re-transplantation (23). In our analysis, the incidence of 42% for overall biliary complications was slightly higher than what is reported in the literature (17, 24).

Acute ischemia-reperfusion injury with associated inflammation leads to severe epithelial damage of bile ducts during and shortly after transplantation. While this is seen in nearly all transplanted livers, regeneration of bile duct epithelium requires a healthy microcirculation in the peribiliary vascular complex and intact peribiliary glands providing progenitor cells for bile duct epithelium. Regeneration requires oxygen therefore, impaired oxygenation due to prolonged ischemia during arterial anastomosis might be of significant influence to the bile duct epithelium with inflammation and fibrogenic reactions. While early bile duct stenosis and leakage is treated interventionally and/or surgically, there is no effective prevention or treatment of non-anastomotic strictures in the long-run despite retransplantation. All of which require constant intervention post-transplant, re-transplantation and are associated with significant morbidity and mortality. In light of an increasing number of extended criteria donation even in DBD livers highly susceptible to biliary complications, there is an urgent need to research preventive techniques. While many studies focus on the impact of DCD donation on biliary complications (15, 16), there is a scarcity of data on risk factors for biliary strictures in DBD-LT, which is the focus of this manuscript.

It should be noted that no DCD organs were used in this cohort. All grafts were obtained from DBD, and neither normothermic regional perfusion nor normothermic machine perfusion NMP was applied. While these techniques are increasingly utilized to improve graft viability, particularly in DCD settings, their absence reflects standard clinical practice during the study period. Given the ongoing discussion around their potential in reducing ischemia-reperfusion injury and associated biliary complications, this represents an important limitation when interpreting the findings.

As our cohort exclusively included grafts from donation after brain death, classical donor warm ischemia time (as defined in DCD protocols) was not applicable.

Furthermore, donor hepatectomy time was not consistently recorded during the study period and could therefore not be included in our analysis. We acknowledge this as a limitation and suggest that future prospective studies incorporate these variables, as they may influence graft viability and postoperative outcomes.

In the present analysis, a trend towards a higher incidence of bile duct strictures (i.e. both non-anastomotic strictures and anastomotic strictures) was found depending on the duration of rWIT. In a meta-analysis of 8,269 liver transplant recipients, hepatic artery thrombosis, longer CIT, longer WIT and total operative times were associated with a higher incidence of biliary strictures (8). Al-Kurd et al. have reported their observation in 1,256 patients of DBD-LTs and demonstrated the positive prognostic impact of rWIT ≤30 min on 1-year and 5-years graft survival (1). Suo et al. presented in their study – including 124 DCD-LT patients (25) – that WIT serves as an independent risk factor for early biliary complications. A recent report by Sakamoto et al. proved in a retrospective single-center study including 67 transplant recipients the association between prolonged rWIT and post-transplant biliary strictures after living-donor LT (20). The authors demonstrated a rWIT >48 min as an independent risk factor for bile duct strictures (p-value 0.008) (20).

On the other hand, although there was a trend towards a higher incidence of bile duct strictures associated with higher serum bilirubin levels on postoperative day five, depending on rWIT, this did not reach statistical significance, possibly due to the small sample size.

The significantly higher bilirubin levels observed on postoperative day 5 in patients with prolonged rWIT likely reflect a more pronounced hepatocellular stress response during the early postoperative period. While transient hyperbilirubinemia is common following liver transplantation, delayed clearance or persistently elevated bilirubin may indicate subclinical ischemia-reperfusion injury or early allograft dysfunction (26, 27). In our cohort, elevated bilirubin levels on day 5 (above the median) were significantly associated with several clinically relevant outcomes, including severe postoperative complications (≥ Clavien-Dindo IIIb), primary nonfunction, biliary complications (such as bile duct strictures), the need for interventional procedures (ERCP or PTCD), as well as prolonged ICU and hospital stays. However, these bilirubin elevations did not correlate with graft survival in our analysis. These findings suggest that bilirubin on day 5 may serve as an early and accessible biomarker of perioperative graft stress and the risk for morbidity, even in the absence of long-term graft loss. Nonetheless, the observed bilirubin elevation may serve as a sensitive, early surrogate marker of initial graft function, particularly in the context of prolonged rWIT.

Interestingly, arterial anastomosis time after portal reperfusion did not correlate with incidence of bile duct strictures. Of note, prolonged arterial anastomosis time may increase ischemic injury to the bile duct, which can lead to necrosis and secondary leaks. Although most biliary complications can be treated, long-term graft and patient survival may be reduced. In our cohort, biliary complications were associated with a higher incidence of primary graft non-function and lower graft survival, ultimately leading to retransplantation, which is consistent with the literature (28). In our cohort, more than 80% of biliary strictures were located at the anastomosis, consistent with prior reports attributing such lesions predominantly to technical factors. These include insufficient arterial blood supply to the bile duct, discrepancies in ductal diameter, and postoperative bile leaks. While prolonged rWIT may contribute to ischemic injury, it is unlikely to represent the sole causative factor in the development of anastomotic strictures. Rather, a multifactorial pathogenesis should be assumed, in which rWIT may exacerbate susceptibility in the presence of technical or anatomic risk factors. It should be considered that in cases with a rWIT >30 min, potential technical difficulties, indicated by increased blood transfusions and longer operative times, may have also influenced graft survival and biliary complications, as well as the need for relaparotomy. Furthermore, graft quality has a significant impact not only on biliary complications but also on acute kidney injury and early allograft dysfunction. Any of these complications can prolong the post-transplant hospital stay and affect both graft and patient survival. In future analysis, the quality of the donor graft and markers of extended criteria donation should be considered. Although we observed a trend in our data, it did not reach statistical significance. Factors such as CIT, graft quality, and the patient’s condition prior to transplant may have influenced our findings. It has been demonstrated that recipient factors such as advanced age, female gender, preoperative hyperbilirubinemia, retransplantation, smoking status, and even socioeconomic status are linked to an increased risk of biliary complications. Furthermore, donor factors, including extended criteria donation, prolonged CIT, split LT, and the use of living donor organs, have also been shown to affect the development of biliary complications (15, 29, 30). With a median labMELD score of 24, our patients were in relatively stable condition, although 13% were listed as high urgent and most patients suffered from various comorbidities not reflected in the MELD score. In all cases, a duct-to-duct anastomosis was performed in an end-to-end fashion, a technique associated with a higher incidence of anastomotic strictures (31). Indeed, 85% of the biliary strictures observed in our cohort were anastomotic stenoses. Therefore, the influence of surgical technique on our results must be considered. Additionally, we acknowledge that the median MELD score differed between the WIT ≤30 min group (17) and the WIT >30 min group (28), although the p-value was 0.067. Our linear regression analysis revealed a statistically significant but weak positive association between the recipient’s MELD score and rWIT (p = 0.0319). However, the explanatory power of the model was low (R^2^ = 0.028), indicating that MELD score alone explains less than 3% of the variability in WIT. This numerical difference suggests that patients with higher MELD scores may have had more severe disease or more complex portal hypertension, potentially leading to increased technical difficulty and prolonged rWIT. Thus, rWIT in our analysis may not only represent an independent ischemic risk factor but also serve as a surrogate marker for surgical complexity and recipient severity.

We therefore caution against interpreting rWIT in isolation.

Although previous studies have suggested a rWIT of ≤30 min as a potential benchmark for improved graft outcomes (1), a definitive threshold effect could not be demonstrated in our cohort. The reference to 30 min in this context should therefore be interpreted as a clinically pragmatic, but not evidence-defined, target. Further studies with larger sample sizes and time-dependent outcome modeling are required to define an optimal cut-off more precisely. Importantly, our analysis focused on recipient WIT rather than cold ischemia time (CIT) or donor WIT, as is often considered in DCD transplantation. Therefore, our findings should not directly impact organ selection or discard decisions.

While it is crucial to arterialize liver perfusion as quickly as possible to mitigate the negative impact of ischemic injury on the bile duct epithelium, other influencing factors should not be overlooked.

## Conclusions

Biliary complications remain a significant cause of postoperative morbidity and the need for retransplantation. While prolonged rWIT may increase the risk of biliary strictures, this effect is likely modulated by multiple donor, graft, and recipient factors. While our analysis identified associations between prolonged rWIT and adverse outcomes such as biliary strictures, primary nonfunction, and elevated bilirubin, these findings must be interpreted with caution.

Due to the retrospective nature of the study, confounding and selection bias—e.g., that technically easier, lower-risk cases tend to have shorter rWIT—cannot be excluded.

Therefore, we refrain from drawing strong causal conclusions and instead propose that rWIT may represent a surrogate for surgical complexity and recipient condition within a broader multifactorial context. Further studies are needed to clarify the impact of each individual factor in order to reduce the incidence and long-term consequences.

## Data Availability

The raw data supporting the conclusions of this article will be made available by the authors, without undue reservation.
