# Supplementary material for: Recipient warm ischemic time negatively influences biliary complications and graft survival – a single center retrospective analysis
Source: Front Gastroenterol (Lausanne). 2025 Aug 6;4:1601741. doi: 10.3389/fgstr.2025.1601741 (PMC12952447; doi:10.3389/fgstr.2025.1601741)
Supplement: Supplementary file 1 [file DataSheet1.pdf]

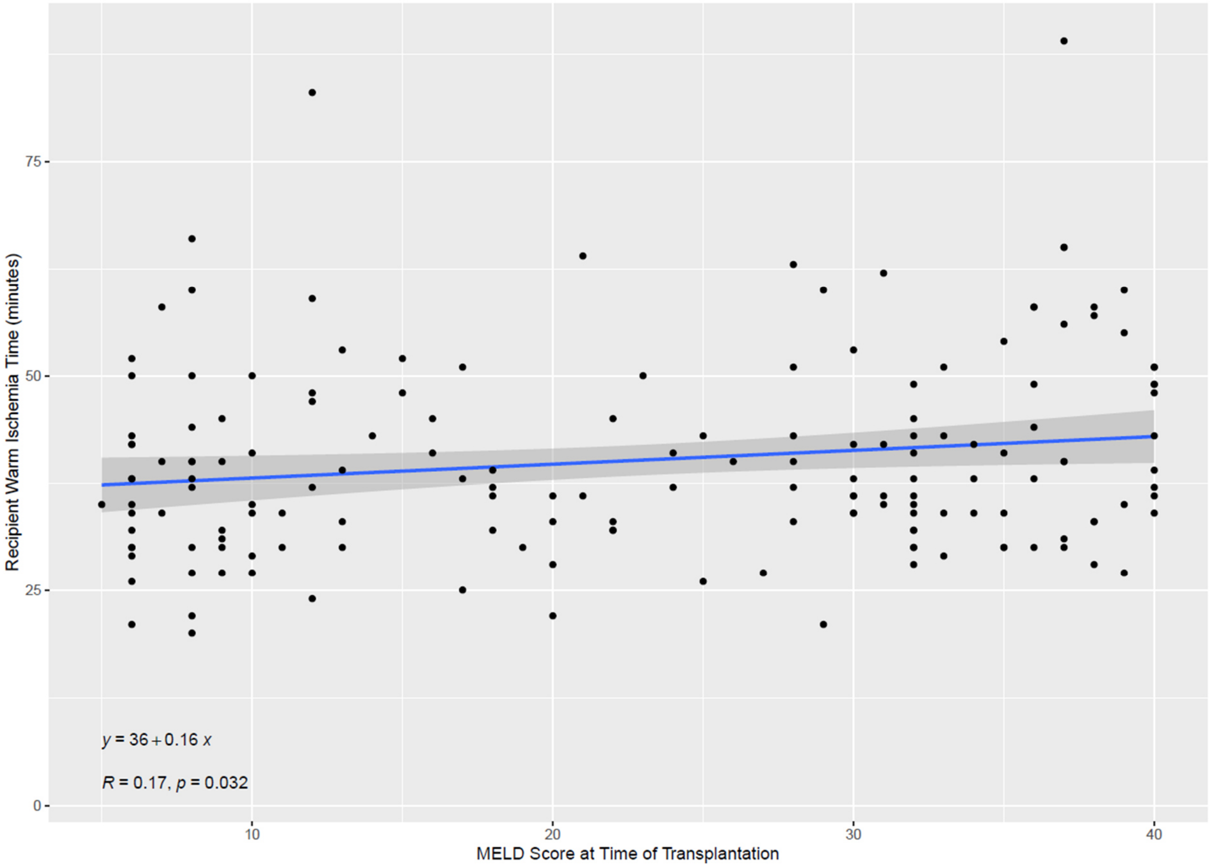

Figure S1 Linear regression analysis showing the relationship between recipient MELD score at time of transplantation and recipient warm ischemia time.

Each point represents an individual transplant case. A slight positive linear trend was observed (β = 0.161, p = 0.0319), but the explained variance was low (R^2 = 0.028), indicating minimal predictive value of MELD score for rWIT.

Abbreviations: MELD-model for end-stage liver disease
